# Endolithic Microbial Life in Extreme Cold Climate: Snow Is Required, but Perhaps Less Is More

**DOI:** 10.3390/biology2020693

**Published:** 2013-04-03

**Authors:** Henry J. Sun

**Keywords:** Antarctica, endolithic microorganisms, snow, cold limit, Mars

## Abstract

Cyanobacteria and lichens living under sandstone surfaces in the McMurdo Dry Valleys require snow for moisture. Snow accumulated beyond a thin layer, however, is counterproductive, interfering with rock insolation, snow melting, and photosynthetic access to light. With this in mind, the facts that rock slope and direction control colonization, and that climate change results in regional extinctions, can be explained. Vertical cliffs, which lack snow cover and are perpetually dry, are devoid of organisms. Boulder tops and edges can trap snow, but gravity and wind prevent excessive buildup. There, the organisms flourish. In places where snow-thinning cannot occur and snow drifts collect, rocks may contain living or dead communities. In light of these observations, the possibility of finding extraterrestrial endolithic communities on Mars cannot be eliminated.

## 1. Introduction

The Ross Desert, an unofficial geographic name referring to high-altitude (>1000 m) areas of the McMurdo Dry Valleys, is one of coldest environments on Earth. Here, the air temperature does not rise much above 0 °C in the peak of summer [1]. The year-round low temperatures create a secondary challenge for life: low water activity, or high aridity. While snow—the only form of precipitation in the region—falls regularly during the summer months, most of the snow is either blown away or sublimates without melting. Together, these two extremes—low temperatures and high aridity—create a desert environment where life is restricted to a few protected niches. Pioneering work by Imre Friedmann and his colleagues showed that the interior of sandstone is one such niche, occupied by cryptoendolithic cyanobacteria and lichens [2,3]. During the summer, the rocks are warmed by solar radiation or insolation, intermittently reaching temperatures high enough to melt snow and support biological activity [1,4,5]. In addition, the sandstones are translucent, especially when wet, with the outer centimeter of the rock, where the organisms reside, receiving 0.1%–1% of incident sunlight [6]. The organisms also actively improve the optical properties of the surrounding sandstone by leaching iron from it [3,7].

The Ross Desert cryptoendoliths do not reside under all available sandstone surfaces, and they don′t survive under all rock surfaces or at all locations. On Mount Fleming, for example, the community is mostly dead and fossilized [8]. Early researchers attributed the absence of life in these locations to the absence of warm temperatures. North-facing slopes, which receive direct solar radiation and are, therefore, warm, are nearly always colonized [3]. In contrast, south-facing slopes, which receive less insolation, are generally devoid of colonization. Taking this logic further, it was suggested that minor changes in temperature during periods of glaciation and global cooling can cause the endolithic community in an entire region to go extinct [7].

In this article, I present an alternative hypothesis, which emphasizes the volume of snow that a rock surface actually receives, or the effective snow condition. In an extreme cold climate where snow is the sole moisture source, photosynthetic microorganisms living within rocks are faced with unique ecological challenges. For instance, snow, unlike rain, cannot wet vertical surfaces. Hence, cliffs are perpetually dry. At the other extreme, a rock can be covered by too much snow. Under a thick snow cover, a rock may no longer receive sufficient insolation to reach temperatures high enough to melt snow. In addition, the light level within the rock may no longer be adequate to support photosynthesis. An ideal effective snow condition occurs on rocks that can trap some snow, but where gravity or frequent gusty winds can prevent excessive buildup. As shown below, all biological variations on Battleship Promontory, which were previously attributed to temperature, can be explained by variations in effective snow condition.

In light of these new observations, the generally-held notion that the surface of Mars is too cold to support extant life [7,8,9,10] should be revisited. Given the recent evidence that suitable rock types, frost formation, and conditions for stable liquid water all occur on Mars, in equatorial lowlands, the possibility of finding living endolithic microorganisms there cannot be eliminated.

## 2. Results and Discussion

### 2.1. Battleship Promontory: Correlation Between Biology and Snow

**Figure 1:**
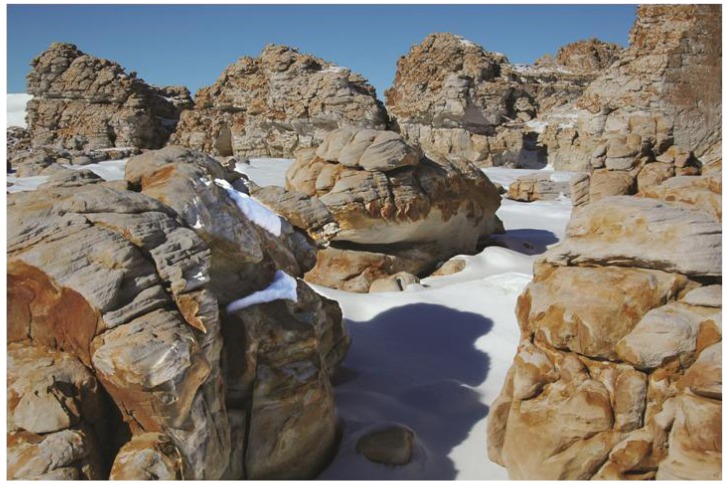
Relationship between rock slope, orientation, and biological activity on Battleship Promontory, Convoy Range, Antarctica. North is to the lower left corner of the photograph (note shadow on the ground). Vertical surfaces are devoid of organisms, as is evident from their dark red coloration, because they cannot trap snow. Sloped surfaces are colonized and show exfoliation, regardless of orientation (boulders in foreground), because they trap some snow, but excess snow is removed by gravity and wind. Heavily-scoured surfaces are devoid of colonization. The rate of scouring is such that the organisms are unable to establish a foothold.

On Battleship Promontory (76°55′S, 161°58′E, elevation 1294 m), in the Convoy Range, sandstone rocks vary widely in size and shape, from outcrops tens of meters across, to boulders a few meters high, and to small stones forming a part of the rubble field (Figure 1). An opportunity to observe the effective snow condition presented itself during a field trip in late January, 2005. Following a significant snowfall, the snow covering the rocks was drastically re-arranged by wind.

Direct contact with snow is not always necessary for a rock to be colonized, and the presence of moisture is not the sole criterion for colonization. For instance, the feet of boulders and stones in loose rubble fields—kept moist by contact with damp soil—are uniformly colonized. Endolithic organisms can also exist within the lower surface of a thin overhang, apparently sustained by downward movement of moisture penetrating the upper surface. In the Dry Valleys, where winds frequently gust up to 15 meters per second, mostly from the southeast [1], some sandstone surfaces are heavily abraded and undergo grain-by-grain disintegration [7]. Under these conditions, slow-growing endolithic organisms are unable to establish a foothold.

These special situations aside, contact with snow is essential for colonization. Hence, vertical cliffs, which cannot trap snow, are devoid of organisms. This is true for both north- and south-facing cliffs (Figure 1). These “abiotic” surfaces are covered by a relatively uniform dark red coating [7]. This coating is the consequence, not the cause, of the rock′s abiotic condition. Where such surfaces have access to moisture, for example, if they lie next to a colonized corner, the coating is destroyed by biological activity and recedes (Figure 1).

**Figure 2:**
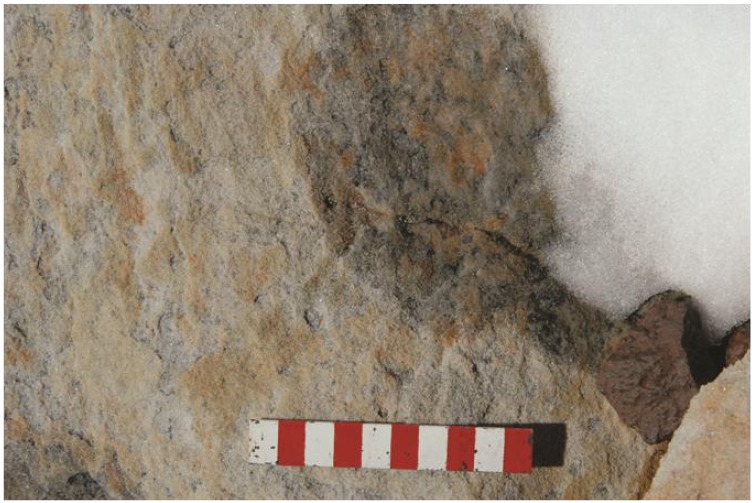
Snow melting on a moderately-sloped boulder top on Battleship Promontory. Melting occurs in summer around noon, when insolation is maximal. (Scale 10 cm).

**Figure 3:**
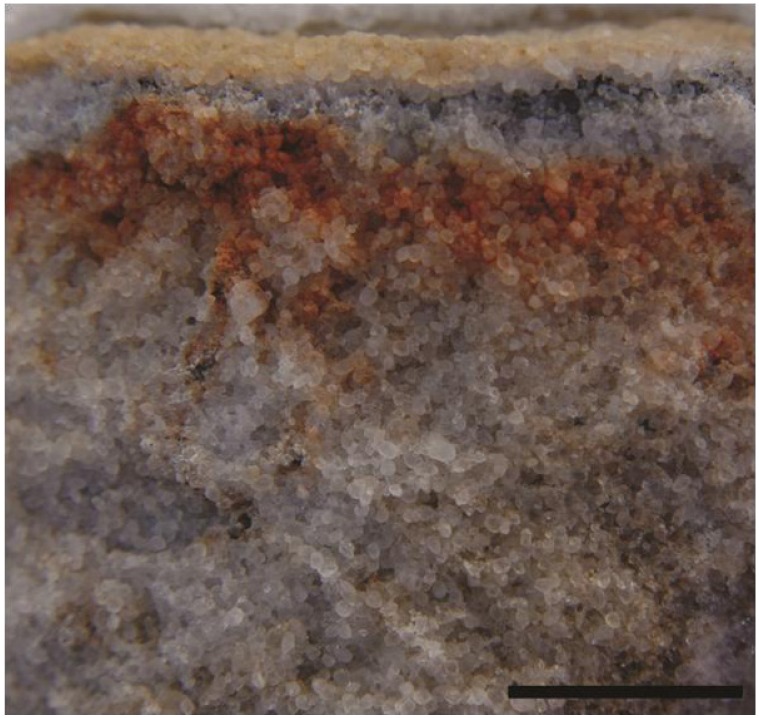
Fractured sandstone showing the presence of microorganisms just below the surface. The leaching of iron from the surrounding sandstone improves photosynthetic access to sunlight. (Scale 5 cm).

**Figure 4:**
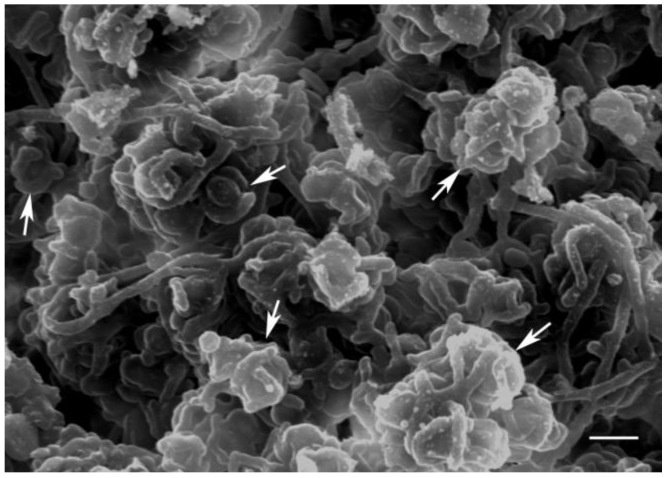
Scanning electron micrograph showing endolithic lichens in sandstone, with soredia, characteristic reproductive structures consisting of small groups of algal cells surrounded by fungal cells (arrows). (Scale 10 µm).

Moderately-sloped surfaces at the tops of boulders have the ideal effective snow condition. They can trap some snow, but excess snow either falls off or is blown away by strong winds. As a result, snow covers on these surfaces are thin, especially around the edges (Figure 2). North- and south-facing slopes are equally well colonized, suggesting that snow, not temperature, controls where the organisms can or cannot exist. The presence of microorganisms under these surfaces was confirmed both in the field (Figure 3) and by examining returned samples using scanning electron microscopy (Figure 4). These relatively dry surfaces are colonized primarily by the lichen-dominated community, while the permanently moist rocks on the ground generally harbor cyanobacteria.

**Figure 5:**
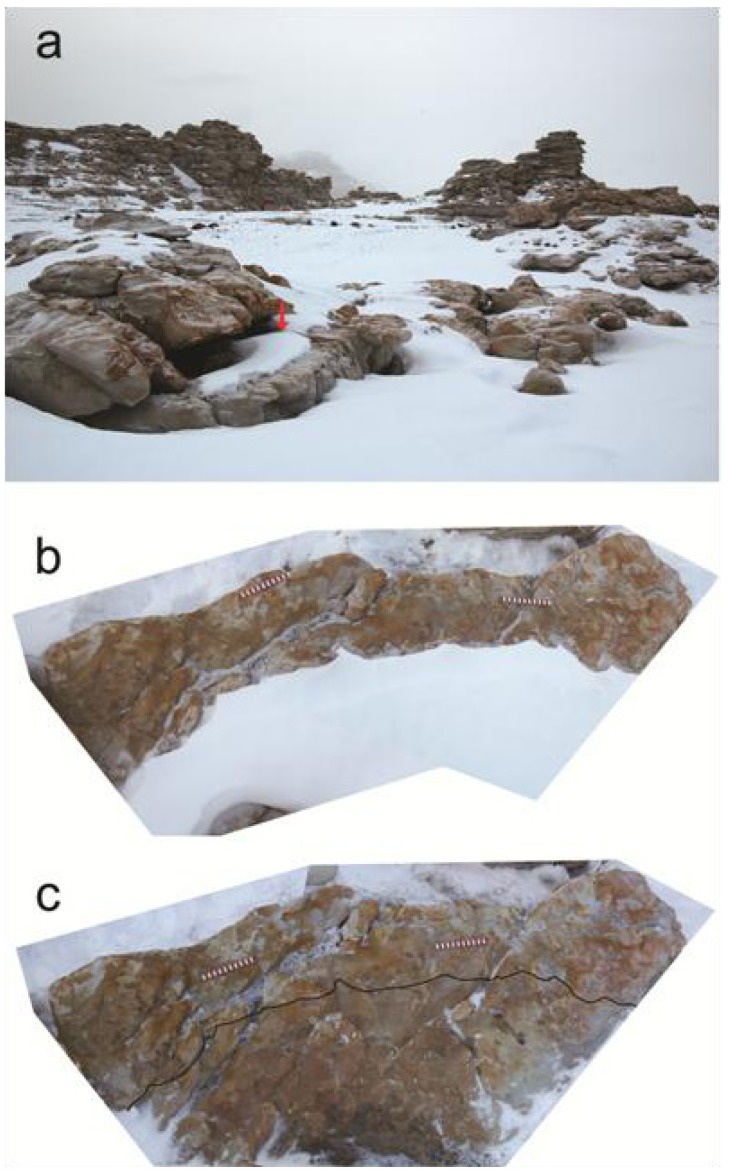
Evidence that persistent thick snow covers are detrimental. (**a**) Partially-covered sandstone surface in the lee of a boulder (red arrow) on Battleship Promontory after winds cleared the snow off the area around the edge. (**b**) View of the exposed area from above, showing that it is heavily colonized. (**c**) View after the snow was deliberately removed, showing that the covered area (below the added black line) is largely devoid of biological activity. (Scale 20 cm).

Perhaps the strongest evidence that snow, not temperature, controls colonization on Battleship Promontory comes from flat, horizontal surfaces. Despite uniform insolation, these surfaces are not always uniformly colonized. Where the colonization is not uniform, it is correlated with the distribution of snow. It seems that the organisms prefer less, not more snow. An example of this observation is shown in Figure 5. This sandstone slab, situated in the lee of a boulder, was partially covered by 8–10 cm of snow (Figure 5a). The photographs in Figure 5b and 5c show a bird’s-eye view of the slab before and after the snow was deliberately removed for observation. The snow-free outer edge is actively colonized, but the snow-covered area shows little evidence of biological activity.

The colonized sandstone rocks on Battleship Promontory can be divided into two categories. First, there are surfaces elevated above the ground. Due to gravity- and wind-assisted snow removal, the effective snow condition of these rocks stays relatively constant and optimal regardless of snowfall volume. These communities appear to be all viable. Second, there are surfaces at ground level and surfaces in a topographic low (e.g., gullies), where snow removal cannot occur and drift snow accumulates. On these surfaces, the effective snow condition can vary considerably and change through time. A surface that is favorable in a climate with low annual precipitation may become unfavorable in a climate with high annual precipitation, and vice versa. In an extreme environment, where the organisms grow slowly [11], extinction occurs relatively quickly, but re-colonization would be slow. As a result, repeated episodes of colonization, death, and re-colonization may occur in these rocks.

### 2.2. Cause of Death on Mount Fleming and Horseshoe Mountain: Climate Cooling or Fluctuations in Precipitation?

Mount Fleming and Horseshoe Mountain, in the Asgard Range, are two sites where sandstone outcrops contain mostly dead and entirely dead microbial communities, respectively. Relative to Battleship Promontory, these sites are located farther south, slightly more inland, and at a higher elevation (2200 m). Accordingly, they have a colder climate. The mean January air temperature on Battleship Promontory is −16.6 °C. In contrast, the values for Mount Fleming and Horseshoe Mountain are −18.7 °C and −22.4 °C, respectively [8]. Based on these data, Friedmann and his colleagues concluded that the cold limit separating hostile from life-supporting environments runs roughly through the area of Mount Fleming [8]. This conclusion was based on the assumption that, at those locations, the communities went extinct because the temperatures no longer rose sufficiently to melt whatever snow there was. In light of the effective snow condition, it is possible that the Mount Fleming and Horseshoe Mountain communities went extinct from changes in annual precipitation, not from low temperature. The landscapes on Mount Fleming and Horseshoe Mountain are relatively flat, with little opportunity to trap snow. During periods of relatively high precipitation, it is possible that exposed surfaces were covered by a thin film of snow that permitted the rocks to warm up to a degree sufficient to produce meltwater. During drier periods with less snow, however, whatever snow there was could have been more effectively removed by the wind, thereby reducing the overall period of metabolic activity and increasing the probability of the death of the community. In this case, the isolated occurrence of colonies on Mount Fleming could be a sign of recovery of the ecosystem during what is now a wetter period.

### 2.3. Extraterrestrial Endolithic Microorganisms on Mars?

**Figure 6:**
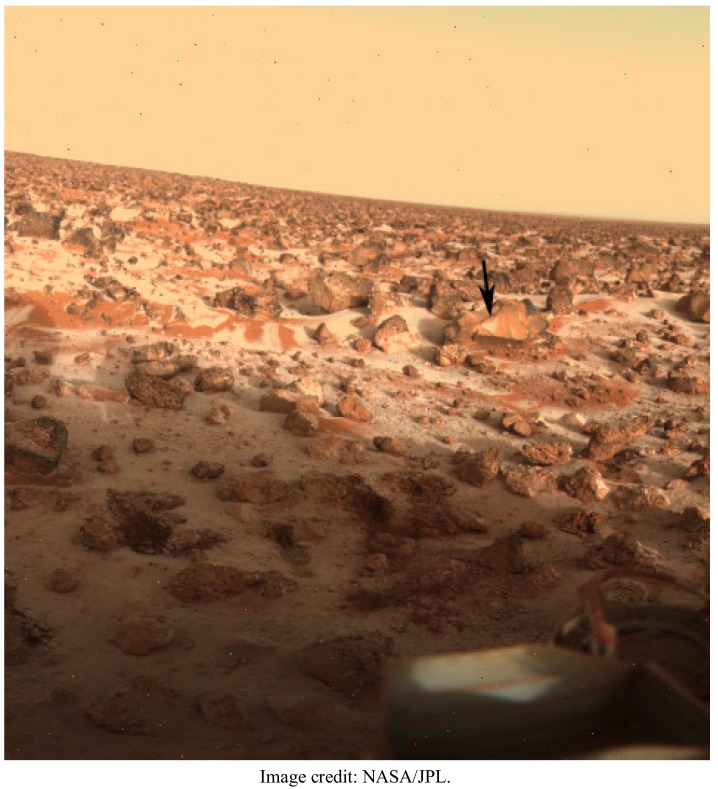
Possible extraterrestrial endolithic habitat on Utopia Planitia, Mars, where water-ice frost formation (arrow), conditions for stable liquid water, and suitable rock types exist.

The surface of Mars is generally considered uninhabitable because of its low atmospheric pressure, somewhat less than 10 mbars. This pressure is below the triple point of water and so does not allow for the presence of stable liquid water. Numerical calculations by Lobitz and colleagues indicate, however, that this may not be the case across the entire planet [12]. Specifically, in low-lying regions between equator and 40°N, temperature and pressure conditions for stable liquid water may occur during summer months. In Utopia Planitia, favorable conditions may last for up to one third of the year. Frost formation in this region is well-documented by images returned by the Viking 2 lander (Figure 6). Furthermore, recent missions indicate that soil sulfate and gypsum, which are suitable for colonization by endolithic organisms [13], are widespread on Mars [14]. Until we definitively establish the cold limit of life on Earth, the possibility that rocks in Utopia Planitia contain live microorganisms cannot be eliminated.

## 3. Experimental Section

Field surveys of biological activity relied on macroscopic biosignatures visible on the rock surface. Where possible, observations were documented by photography. The following protocol was used to prepare samples for electron microscopy. Specimens were rehydrated in saline phosphate buffer and then fixed in 0.5% formaldehyde and 1% glutaraldehyde for 15 minutes. Fixed specimens were dehydrated in an ethanol series: 15%, 30%, 50%, 75%, 95%, 100%, each for 15 minutes. After two additional changes in anhydrous ethanol, the specimens were placed in hexamethyldisilazane (HMDS) twice, each time for 30 minutes. After the second wash, the HMDS was decanted, and the specimens were air-dried. Specimens were carbon-coated and viewed using a scanning electron microscope (JSM-5610).

## 4. Conclusions

On Battleship Promontory, colonization barriers of microbial communities under sandstone surfaces are imposed by the uneven distribution of snow, not temperature. The presence of dead communities under some rock surfaces may be attributed to fluctuations in annual precipitation. On Mount Fleming and Horseshoe Mountain, the communities may have died during a period of climatic cooling or during a period when changes in annual precipitation caused the effective snow condition to become unfavorable. On Mars, extant endolithic communities may exist in equatorial lowlands.
